# Effects of Selenium Supplementation on the Ion Homeostasis in the Reproductive Organs and Eggs of Laying Hens Fed With the Diet Contaminated With Cadmium, Lead, Mercury, and Chromium

**DOI:** 10.3389/fvets.2022.902355

**Published:** 2022-06-10

**Authors:** Caimei Wu, L. Li, Y. X. Jiang, Woo Kyun Kim, B. Wu, G. M. Liu, Jianping Wang, Y. Lin, K. Y. Zhang, J. P. Song, R. N. Zhang, F. L. Wu, K. H. Liang, Shiping Bai

**Affiliations:** ^1^Key Laboratory for Animal Disease-Resistance Nutrition and Feedstuffs of China Ministry of Agriculture and Rural Affairs, Institute of Animal Nutrition, Sichuan Agricultural University, Chengdu, China; ^2^Department of Poultry Science, University of Georgia, Athens, GA, United States; ^3^Chelota biotechnology CO., LTD, Deyang, China; ^4^Institute of Food and Nutrition Development, Ministry of Agriculture and Rural Affairs, Beijing, China

**Keywords:** selenium, heavy metals, laying hens, trace elements, disorder

## Abstract

The objective of this study was to explore the toxic effects of different heavy metals in combination with their deposition and ion homeostasis in the reproductive organs and eggs of laying hens, as well as the alleviating action of selenized yeast. A total of 160 Lohmann pink-shell laying hens (63-week-old) were randomly allocated into four treatments with 10 replicates of four hens each. The four dietary treatments were the corn-soybean meal basal dietary (control; **CON**); the CON dietary supplemented with 0.4 mg/kg selenium from selenized yeast (**Se**); the CON dietary supplemented with 5 mg/kg Cd + 50 mg/kg Pb +3 mg/kg Hg + 5 mg/kg Cr (**HEM**), and the HEM dietary supplemented with 0.4 mg/kg selenium from selenized yeast (**HEM+Se**). The dietary HEM significantly increased Cd, Pb, and Hg deposition in the egg yolk and ovary, and Cd and Hg deposition in the oviduct and in the follicular wall (*p* < 0.05). The HEM elevated Fe concentration in the egg yolk, ovary, and oviduct (*p* < 0.05). The HEM decreased Mn concentration in the egg yolk, Fe, Mn, and Zn concentrations in the egg white, Cu concentration in the ovary, Mg concentration in the oviduct, as well as Ca, Cu, Zn, and Mg concentrations in the follicular walls (*p* < 0.05). Dietary Se addition elevated Se concentration in the egg yolk, oviduct, and follicular walls and Mg concentration (*p* < 0.05) in the oviduct, whereas it reduced Fe concentration in the oviduct compared with the HEM-treated hens. Some positive or negative correlations among these elements were observed. Canonical Correlation Analysis showed that the concentrations of Pb and Hg in the egg yolk were positively correlated with those in the ovary. The concentration of Cd in the egg white was positively correlated with that in the oviduct. In summary, dietary Cd, Pb, Hg, and Cr in combination caused ion loss and deposition of HEM in reproductive organs of laying hens. Dietary Se addition at 0.4 mg/kg from selenized yeast alleviated the negative effects of HEM on Fe and Mg ion disorder in the oviduct and follicle wall of hens.

## Introduction

Heavy metal contamination in animal feed has received much attention in recent years due to intensive agricultural development. The cadmium (Cd), mercury (Hg), and lead (Pb) are considered contaminants in poultry feed or diet, which are a prior hazard to animal health because they can induce organ damage by oxidative stress, even at the low exposure levels (1, 2). Excessive exposure to a higher concentration of chromium (Cr) is linked with cellular or systemic disorders (3, 4), although Cr is essential to maintain various biochemical and physiological functions in animals (2). In poultry, dietary Pb and Cd contamination in combination damaged the liver by oxidative stress in laying hens (5, 6). Previous studies found that dietary heavy metals contamination increased their concentration in different tissues of poultry, especially the reproductive organs or tissues (7–10). Excessive Cd changed the balance of metallic ions in the muscle, kidney, liver, and ovary of chickens (12–14), which is vital for life as some metallic ions serve as cofactors for many different proteins (15). However, few studies have focused on the effects of different heavy metals in combination on the metallic ions in the different reproductive organs or tissue in chickens.

Selenium is an essential micronutrient for poultry (16), and can counteract the toxicity of heavy metals such as Cd and inorganic Hg (17, 18). Previous studies have found that dietary Se supplementation decreased the deposition of Cd or Pb in different organs of laying hens (12, 19). Dietary Se addition could relieve the Cd or Pb-caused ion disorders in the kidneys, ovary, muscle, and liver of chicken (12–14, 19, 20). However, few researchers have addressed the effects of dietary Se addition on the metallic ions concentration in reproductive organs of laying hens fed several heavy metals contaminated diet.

The maximum permitted limits of Cd, Pb, Hg, and Cr are 0.5, 5, 0.1, and 5 mg/kg, respectively, in the complete poultry feed according to the guideline of China (21). Several reports showed that the concentration of Cd, Pb, or Hg in the complete poultry feed has a large range from non-detectable to 150, 15, or 20-fold of the maximum permitted concentration, respectively (10, 22, 23). The heavy metals toxicity study found that the Cd concentrations up to 5 mg/kg, the Pb concentrations up to 20 mg/kg when dietary calcium levels are high, and the Hg and Cr concentrations up to 3 mg/kg are most likely to start gross clinical symptoms of poultry (24). Dietary addition of 3 mg/kg Cr negative affected the laying performance of hens (25) and the growth performance of broilers (4). Our previous study found that dietary contamination with 5 mg/kg Cd, 50 mg/kg Pb, 3 mg/kg Hg, and 5 mg/kg Cr has a negative impact on the laying performance and egg quality of hens in late-laying period (26). Meanwhile, the maximum permitted limit of Se in poultry feed is 0.5 mg/kg, based on the Se content of 1.4 mg/kg in basal dietary, 0.4 mg/kg Se from selenized yeast was used in this study. Therefore, the objective of this study was to explore the toxic effects of different heavy metals in combination with their deposition and ion homeostasis in the reproductive organs and eggs of laying hens, as well as the alleviating action of selenized yeast.

## Materials and Methods

### Materials

Cadmium chloride (CdCl_2_), lead nitrate (Pb(NO_3_)_2_), mercury bichloride (HgCl_2_), and chromium trichloride (CrCl_3_) were purchased from Kelong company of Chengdu (Sichuan, China). Selenized yeast (SY) which contains 0.2% selenium was provided by Chelota biotechnology Co. Ltd (Deyang Guanghan, Sichuan, China). The standard solution of Cd, Cr, Pb, Hg, Fe, Cu, Mn, Zn, Na, K, Mg, Se, and Ca were purchased from the center of the national standard. Laying hens were Lohmann pink-shell with similar production performance obtained from commercial layer farms (Mianyang, Sichuan, China).

### Experimental Design

A total of 160 healthy Lohmann pink-shell laying hens (63-wk-old) were randomly allocated into four treatments with 10 replicates of four hens. Two adjacent cages (two birds each cage) were used as one replicate unit. The four dietary treatments were as follows: the corn-soybean meal basal diet (control; **CON**), the CON diet supplemented with 0.4 mg/kg selenium from selenized yeast (**Se**), the CON diet added with 5 mg/kg Cd, 50 mg/kg Pb, 3 mg/kg Hg, and 5 mg/kg Cr (**HEM**), and the HEM diet supplemented with 0.4 mg/kg Se (**HEM+Se**). The experiment lasted 12 weeks. The corn-soybean meal basal diet (metabolizable energy 10.90 MJ/kg, crude protein 14.56 %, calcium 3.55 %, and nonphytate phosphorus 0.33 %) was formulated to meet or exceed the requirement of laying hens (27). The analyzed concentrations of Cd, Cr, Pb, Hg, Fe, Cu, Mn, Zn, Na, K, Mg, Ca, and Se in different experimental diets are presented in Table 1. All hens were raised in a thermostatically controlled house (24 ± 1.0°C), with *ad libitum* access to feed and water. A photoperiod of 16 h light and 8 h dark was maintained.

**Table 1:** The analyzed values of Cd, Cr, Pb, Hg, Fe, Cu, Mn, Zn, Na, K, Mg, Ca, and Se for all diets and critical components of basal diet (air-dry basis).

**Item^**a**^**	**Cd^**b**^ (mg/kg)**	**Cr^**b**^ (mg/kg)**	**Pb^**b**^ (mg/kg)**	**Hg^**b**^ (mg/kg)**	**Se^**b**^ (mg/kg)**	**Cu^**b**^ (mg/kg)**	**Fe^**b**^ (mg/kg)**	**Mn^**b**^ (mg/kg)**	**Zn^**b**^ (mg/kg)**	**Na^**b**^ (%)**	**K^**b**^ (%)**	**Mg^**b**^ (%)**	**Ca^**b**^ (%)**
CON	0.18 ± 0.06	7.9 ± 0.27	2.11 ± 0.16	0.01 ± 0.0009	0.14 ± 0.004	14.78 ± 3.03	182.02 ± 26.98	63.12 ± 3.67	98.83 ± 16.81	0.27 ± 0.09	2.14 ± 0.33	0.31 ± 0.058	3.84 ± 0.42
Se	0.29 ± 0.05	6.23 ± 0.21	1.49 ± 0.42	0.01 ± 0.0008	0.51 ± 0.02	15.56 ± 0.84	179.17 ± 14.21	69.13 ± 5.66	97.46 ± 6.60	0.22 ± 0.034	2.11 ± 0.20	0.33 ± 0.029	4.19 ± 0.69
HEM	5.25 ± 0.2	13.31 ± 0.41	52.84 ± 2.95	3.18 ± 0.67	0.15 ± 0.01	12.62 ± 0.49	175.71 ± 11.51	66.81 ± 11.33	90.32 ± 23.87	0.26 ± 0.043	1.86 ± 0.41	0.25 ± 0.20	5.15 ± 0.27
HEM+Se	4.97 ± 0.09	12.69 ± 0.64	53.76 ± 2.38	3.29 ± 0.14	0.52 ± 0.01	12.94 ± 0.69	195.88 ± 10.00	69.78 ± 10.08	99.75 ± 15.15	0.25 ± 0.030	2.05 ± 0.089	0.27 ± 0.0079	4.50 ± 0.33

a *CON, Corn-Soybean Meal Basal Dietary; Se, the Basal Dietary Supplemented With 0.4 mg/kg Selenium From Selenized Yeast (the Selenium Concentration Was 0.14 mg/kg in the CON Diet); HEM, the Basal Dietary Supplemented With 5 mg/kg Cadmium From CdCl_2_, 50 mg/kg Lead From Pb(NO_3_)_2_, 3 mg/kg Mercury From HgCl_2_ and 5 mg/kg Chromium From CrCl_3_; HEM+Se, the HEM Dietary Supplemented With 0.4 mg/kg Selenium From Selenized Yeast*.

b *Cd, cadmium; Cr, chromium; Pb, lead; Hg, mercury; Se, selenium; Cu, copper; Fe, iron; Mn, manganese; Zn, zinc; Na, sodium; K, potassium; Mg, magnesium; Ca, calcium*.

### Sample Collection

At the end of the experiment, the egg yolk and egg white of every three eggs from each replicate were pooled into one sample for the mineral analyses (10 egg yolk samples and 10 egg white samples/treatment). Two birds were selected from each replicate and euthanized by cervical dislocation, and the samples of the ovary and oviduct were collected for the minerals analyses. And then, the follicular walls of the largest yellow follicles with a diameter ranging from 38 to 42 mm, also known as late preovulatory follicles prior to ovulation were also collected and stored at −20°C until the minerals analyses.

### Determination of Mineral Elements

Egg yolks and egg whites were lyophilized prior to analyses. Then, a 0.5 g sample of lyophilized egg yolk or egg white, or ovary, oviduct, or follicular wall on the wet-tissue basis was transferred into a polytetrafluoroethylene digestion vessel. Then, 10 ml of HNO_3_/HClO_4_ (4:1, v/v) was added to the vessel and the digestion was carried out at 180°C using a heater (Model BHW-09Y, Botonyc, Beijing, China). After digestion, the residual solution (about 0.2 ml) was dissolved with 0.5 % HNO_3_ solution in a certain volumetric flask to obtain the sample solution. This sample solution was directly used for measuring the concentrations of Cd, Cr, Pb, Hg, Fe, Cu, Mn, and Zn. For determining the concentrations of Na, K, Mg, and Ca, the lanthanum oxide (10 mg/L, m/v) and cesium chloride (10 mg/L, m/v) were added to the sample solution. The concentrations of the Fe, Cu, Mn, Zn, Cd, Pb, and Cr were determined using an atomic absorption spectrometer (Contr AA700, Analytik Jena AG, Jena, Germany) according to the China National Standard GB/T 13885-2017 (28). The concentration of Hg was measured using a mercury determination instrument (DMA-80 Evo, Milestone, Italy) according to the China National Standard GB 5009.17-2021 (29). The lanthanum oxide (purity 99.99%) and chloride cesium (purity 99.99%) were purchased from Sangon Biotech Company (Shanghai, China). The instrument detection limits of Fe, Cu, Mn, Zn, Cd, Pb, Cr, Na, K, Mg, and Ca were 0.05, 0.02, 0.02, 0.02, 0.01, 0.01, 0.02, 0.02, 0.02, 0.01, and 0.02 mg/kg, respectively. The instrument detection limit of Hg was 0.50 μg/kg. The concentration of Se was measured using a dual-channel atomic fluorescence photometer (AFS-230E, Haiguang Instrument, Beijing, China) according to China National Standard GB 5009.93-2017 (31). In brief, 1.0 g of the above tissue sample was digested with HNO_3_/HClO_4_ (4:1, v/v) at 180°C using a heater (Model BHW-09Y, Botonyc, China), and the residual solution was dissolved with 4.73 M HCl containing 200 g/L (m/v) of potassium ferricyanide for Se analysis. The instrument detection limit of Se was 0.30 μg/kg. For quality control, a standard reference of wheat powder or bovine liver provided by the National Institute of Standards and Technology (Beijing, China) was included in each batch of analysis to verify the determination validation. The R-squared values of standard curves for the different minerals were higher than 0.99. The recovery rates of Fe, Cu, Mn, Zn, Cd, Pb, Cr, Na, K, Mg, Ca, Hg, and Se were 90.1–99.5%, 93.8–102.3%, 90.9–103.4%, 95.3–105.4%, 91.8–98.4%, 93.9–97.4%, 96.9–100.3%, 97.3–104.1%, 93.8–102.4%, 94.5–102.6%, 96.3–105.4%, 97.9–103.7%, and 92.9–102.1%, respectively.

### Statistical Analysis

The PROC MIXED procedure of SAS software (version 9.4, SAS Inst. Inc., Cary, NC, USA) was used to analyze the data about concentrations of Cd, Cr, Pb, Hg, Se, Fe, Cu, Mn, Zn, Na, K, Mg, and Ca in the egg yolk, egg white, ovary, oviduct, and follicular walls.

The model is as follows:


$$\begin{array}{l} Y_{\text{ij}}=\mu +{\text{α}}_{\text{i}}+{\text{ε}}_{\text{ij}} \end{array}$$

Y is an observation of the dependent variable, μ is the population mean for the variable, α_i_ is the fixed effect of treatment (CON, Se, HEM, or HEM+Se), ε_ij_ is the random error associated with the observation. Each replicate was used as the experimental unit. The Tukey test was used for multiple population comparisons among the different treatments with letter groupings. The results are presented as the mean and standard error of the mean (SEM). Values of *p* < 0.05 were considered statistically significant. Correlations between elements were investigated using Pearson's correlation test.

Additionally, Canonical Correlation Analysis (CCA) was used to analyze correlations between heavy metals in the egg yolk and ovary, as well as egg white and oviduct, *r* > 0.50 and *p* < 0.05 were considered as an indication of a strong correlation.

CCA is a multivariate statistical analysis method that reflects the overall correlation between two sets of data objects by using the correlation between comprehensive variable pairs. Its basic principle is that in order to overall analyze the relevant relations between the two sets of data objects, typical two variables V_1_ and W_1_ were extracted in the two groups of variables, respectively. V_1_ and W_1_ were the linear combinations of the variables for the two variables group, respectively. The overall correlation between the two sets of data objects was reflected by using the relationship between two comprehensive variables (30).

## Results

### The Deposition of Cd, Pb, Hg, Cr and Ion Concentration in the Reproductive Organs and Eggs

#### Egg Yolk

**Figure 1:**
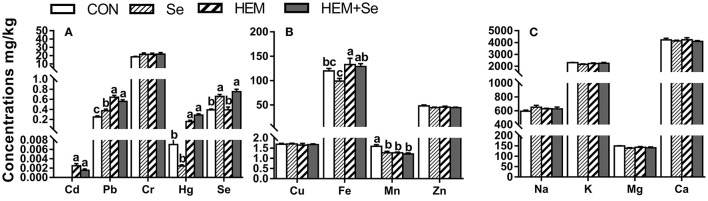
The effect of selenium and cadmium, lead, mercury, and chromium Co-treatment on the concentrations of ions in the egg yolk (Wet Basis mg/kg) of laying hens from 63 to 74 wk of age. CON, corn-soybean meal basal dietary; Se, the basal dietary supplemented with 0.4 mg/kg selenium from selenized yeast (the selenium concentration was 0.14 mg/kg in the CON diet); HEM, the basal dietary supplemented with 5 mg/kg cadmium from CdCl_2_, 50 mg/kg lead from Pb(NO_3_)_2_, 3 mg/kg mercury from HgCl_2_ and 5 mg/kg chromium from CrCl_3_; HEM+Se, the HEM dietary supplemented with 0.4 mg/kg selenium from selenized yeast. **(A)** The effect of selenium and cadmium, lead, mercury, and chromium Co-treatment on the concentrations of cadmium, lead, chromium, mercury, and selenium in the egg yolk. **(B)** The effect of selenium and cadmium, lead, mercury, and chromium Co-treatment on the concentrations of copper, iron, manganese, and zinc in the egg yolk. **(C)** The effect of selenium and cadmium, lead, mercury, and chromium Co-treatment on the concentrations of sodium, potassium, magnesium, calcium in the egg yolk. ^a,b,c^ Bars without a shared common letter are significantly different (*p* < 0.05). Data show the mean ± SEM (*n* = 10).

Dietary HEM significantly increased (*p* < 0.05) the concentrations of Cd, Pb, Hg, and Fe (Figure 1), but significantly decreased (*p* < 0.05) the concentration of Mn in the egg yolk compared to the CON. Decreased as down in Figure 1A. Dietary treatments did not significantly affect the concentrations of Cu, Zn, Na, K, Mg, and Ca in the egg yolk (*p* > 0.05). However, the HEM diet supplemented with SY increased (*p* < 0.05) the concentration of Se, did not significantly affect the concentrations of Cd, Pb, Hg, Fe, and Mn in the egg yolk (*p* > 0.05) compared to the HEM-treated group.

#### Egg White

**Figure 2:**
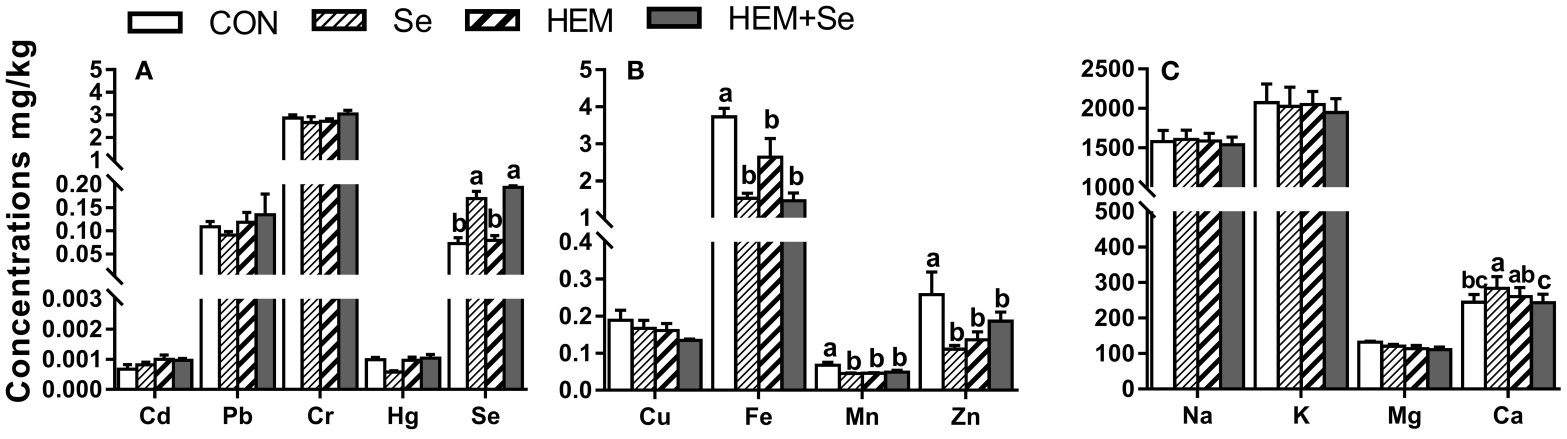
The effect of selenium and cadmium, lead, mercury, chromium Co-treatment on the concentrations of ions in the egg white (Wet Basis mg/kg) of laying hens from 63 to 74 wk of age. CON, corn-soybean meal basal dietary; Se=the basal dietary supplemented with 0.4 mg/kg selenium from selenized yeast (the selenium concentration was 0.14 mg/kg in the CON diet); HEM, the basal dietary supplemented with 5 mg/kg cadmium from CdCl_2_, 50 mg/kg lead from Pb(NO_3_)_2_, 3 mg/kg mercury from HgCl_2_ and 5 mg/kg chromium from CrCl_3_; HEM+Se, the HEM dietary supplemented with 0.4 mg/kg selenium from selenized yeast. **(A)** The effect of selenium and cadmium, lead, mercury, chromium Co-treatment on the concentrations of cadmium, lead, chromium, mercury, selenium in the egg white. **(B)** The effect of selenium and cadmium, lead, mercury, chromium Co-treatment on the concentrations of copper, iron, manganese, zinc in the egg white. **(C)** The effect of selenium and cadmium, lead, mercury, chromium Co-treatment on the concentrations of sodium, potassium, magnesium, calcium in the egg white. ^a,b,c^ Bars without a shared common letter are significantly different (*p* < 0.05). Data show the mean ± SEM (*n* = 10).

Dietary HEM significantly reduced (*p* < 0.05) the concentrations of Fe, Mn, and Zn compared to the CON. Whereas the HEM diet supplemented with SY significantly reduced (*p* < 0.05) the accumulation of Ca in the egg white compared with the HEM treatment (Figure 2). Dietary treatments did not significantly affect the concentrations of Cd, Pb, Cr, Hg, Cu, Na, K, and Mg in the egg white (*p* > 0.05).

#### Ovary

**Figure 3:**
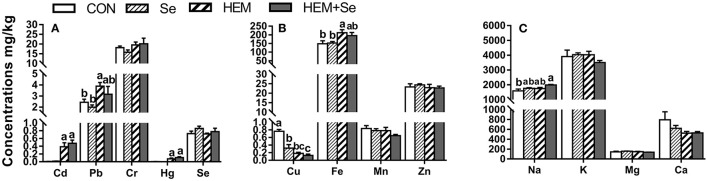
The effect of selenium and cadmium, lead, mercury, chromium Co-treatment on the concentrations of ions in the ovary (Wet Basis mg/kg) of laying hens from 63 to 74 wk of age. CON, corn-soybean meal basal dietary; Se, the basal dietary supplemented with 0.4 mg/kg selenium from selenized yeast (the selenium concentration was 0.14 mg/kg in the CON diet); HEM, the basal dietary supplemented with 5 mg/kg cadmium from CdCl_2_, 50 mg/kg lead from Pb(NO_3_)_2_, 3 mg/kg mercury from HgCl_2_ and 5 mg/kg chromium from CrCl_3_; HEM+Se, the HEM dietary supplemented with 0.4 mg/kg selenium from selenized yeast. **(A)** The effect of selenium and cadmium, lead, mercury, chromium Co-treatment on the concentrations of cadmium, lead, chromium, mercury, selenium in the ovary. **(B)** The effect of selenium and cadmium, lead, mercury, chromium Co-treatment on the concentrations of copper, iron, manganese, zinc in the ovary. **(C)** The effect of selenium and cadmium, lead, mercury, chromium Co-treatment on the concentrations of sodium, potassium, magnesium, calcium in the ovary. ^a,b,c^ Bars without a shared common letter are significantly different (*p* < 0.05). Data show the mean ± SEM (*n* = 10).

Dietary HEM significantly increased (*p* < 0.05) the concentrations of Cd, Hg, Pb, and Fe, but significantly reduced (*p* < 0.05) the concentration of Cu in the ovary compared to the CON (Figure 3). However, the HEM diet supplemented with SY had no effect (*p* > 0.05) on the concentrations of Cd, Pb, Hg, Fe, and Cu compared to the HEM-treated group. Dietary treatments did not significantly affect the concentrations of Cr, Se, Mn, Zn, K, Mg, and Ca in the ovary (*p* > 0.05).

#### Oviduct

**Figure 4:**
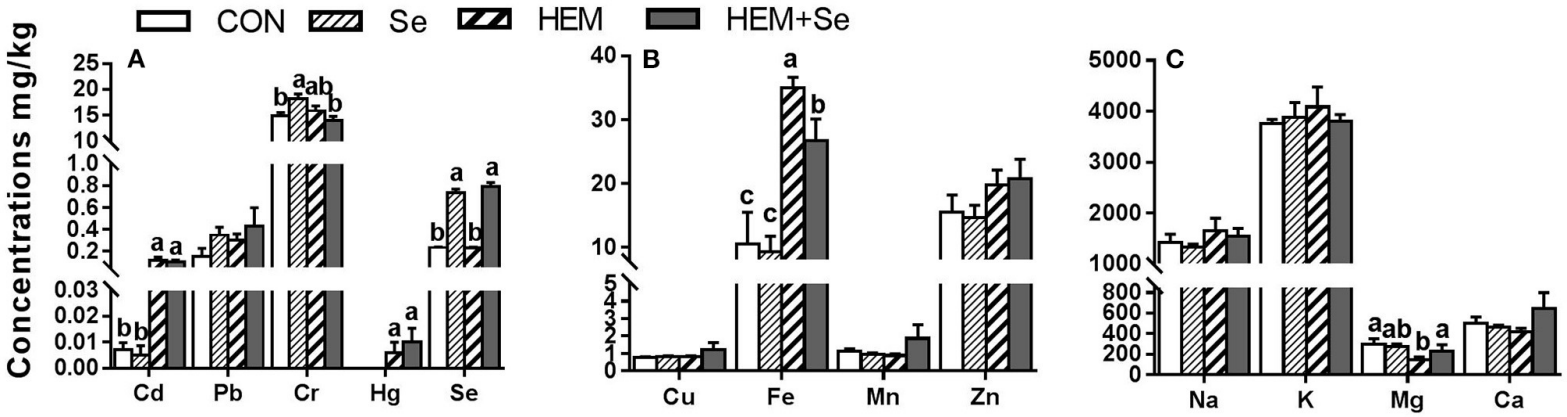
The effect of selenium and cadmium, lead, mercury, chromium Co-treatment on the concentrations of ions in the oviduct (Wet Basis mg/kg) of laying hens from 63 to 74 wk of age. CON, corn-soybean meal basal dietary; Se, the basal dietary supplemented with 0.4 mg/kg selenium from selenized yeast (the selenium concentration was 0.14 mg/kg in the CON diet); HEM, the basal dietary supplemented with 5 mg/kg cadmium from CdCl_2_, 50 mg/kg lead from Pb(NO_3_)_2_, 3 mg/kg mercury from HgCl_2_ and 5 mg/kg chromium from CrCl_3_; HEM+Se, the HEM dietary supplemented with 0.4 mg/kg selenium from selenized yeast. **(A)** The effect of selenium and cadmium, lead, mercury, chromium Co-treatment on the concentrations of cadmium, lead, chromium, mercury, selenium in the oviduct. **(B)** The effect of selenium and cadmium, lead, mercury, chromium Co-treatment on the concentrations of copper, iron, manganese, zinc in the oviduct. **(C)** The effect of selenium and cadmium, lead, mercury, chromium Co-treatment on the concentrations of sodium, potassium, magnesium, calcium in the oviduct. a, b, c Bars without a shared common letter are significantly different (*p* < 0.05). Data show the mean ± SEM (*n* = 10).

Dietary HEM significantly elevated (*p* < 0.05) the concentrations of Cd, Hg, and Fe compared to the CON, whereas it decreased (*p* < 0.05) the concentration of Mg in the oviduct compared to the CON (Figure 4). The HEM diet supplemented with SY significantly reduced (*p* < 0.05) the concentration of Fe and significantly increased the concentrations of Mg and Se (*p* < 0.05), and had no significant effect (*p* > 0.05) on the accumulations of Cd and Hg compared to the HEM-treated group. Dietary treatments also did not significantly affect the concentrations of Cu, Mn, Zn, Na, K, and Ca in the oviduct (*p* > 0.05).

#### Follicular Walls

**Figure 5:**
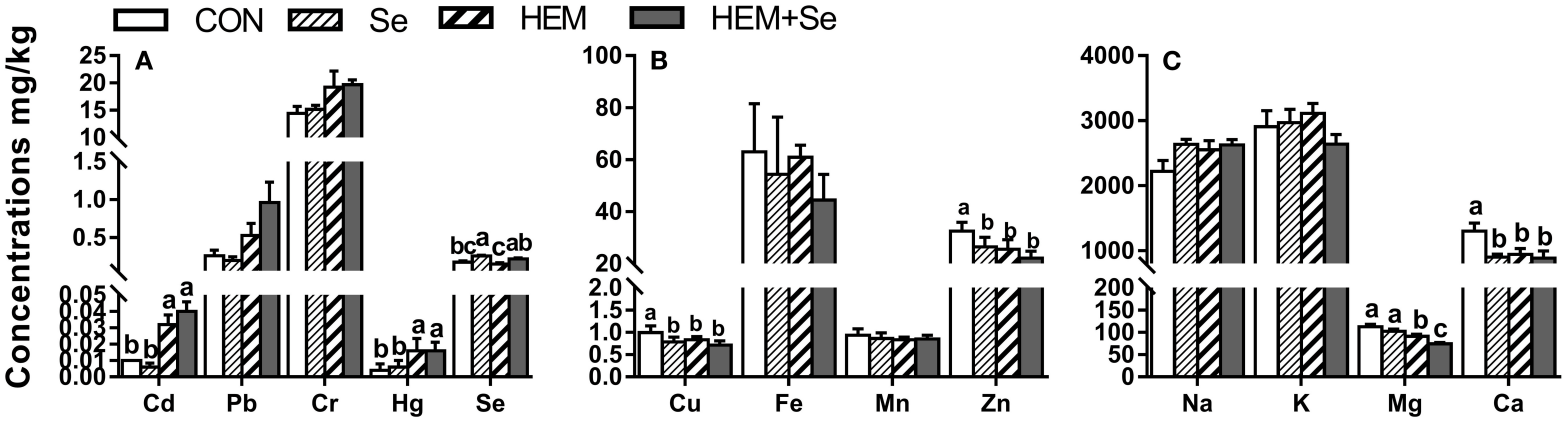
The effect of selenium and cadmium, lead, mercury, chromium Co-treatment on the concentrations of ions in the follicular walls (Wet Basis mg/kg) of laying hens from 63 to 74 wk of age. CON, corn-soybean meal basal dietary; Se, the basal dietary supplemented with 0.4 mg/kg selenium from selenized yeast (the selenium concentration was 0.14 mg/kg in the CON diet); HEM, the basal dietary supplemented with 5 mg/kg cadmium from CdCl_2_, 50 mg/kg lead from Pb(NO_3_)_2_, 3 mg/kg mercury from HgCl_2_ and 5 mg/kg chromium from CrCl_3_; HEM+Se, the HEM dietary supplemented with 0.4 mg/kg selenium from selenized yeast. **(A)** The effect of selenium and cadmium, lead, mercury, chromium Co-treatment on the concentrations of cadmium, lead, chromium, mercury, selenium in the follicular walls. **(B)** The effect of selenium and cadmium, lead, mercury, chromium Co-treatment on the concentrations of copper, iron, manganese, zinc in the follicular walls. **(C)** The effect of selenium and cadmium, lead, mercury, chromium Co-treatment on the concentrations of sodium, potassium, magnesium, calcium in the follicular walls. ^a,b,c^ Bars without a shared common letter are significantly different (*p* < 0.05). Data show the mean ± SEM (*n* = 10).

Dietary HEM increased (*p* < 0.05) the accumulations of Cd and Hg, but reduced (*p* < 0.05) the concentrations of Cu, Zn, Ca, and Mg in the follicular walls compared to the CON (Figure 5). The HEM diet supplemented with SY significantly elevated Se concentration but reduced (*p* < 0.05) the concentration of Mg, and had no effect (*p* > 0.05) on the concentrations of Cd, Hg, Cu, Zn, and Ca in the follicular walls compared to the HEM-treated group. Dietary treatments also did not significantly affect the concentrations of Pb, Cr, Fe, Mn, Na, and K in the follicular walls (*p* > 0.05).

### Correlation Coefficients Among Some Elements in the Reproductive Organs and Eggs

#### Egg Yolk

There were 13 significant correlations among the concentration of 13 elements in the egg yolk (Table 2). The element Cd was positively correlated with Pb (*p* < 0.01), Hg (*p* < 0.05), and Fe (*p* < 0.05). Pb was positively correlated with Cd (*p* < 0.01) and negatively correlated with Mn (*p* < 0.01). Hg was positively correlated with Cd (*p* < 0.05) and Cr (*p* < 0.05). Se was negatively correlated with Mn (*p* < 0.05). Cu was positively correlated with Zn (*p* < 0.01) and Ca (*p* < 0.01). Zn was positively correlated with Cu (*p* < 0.01), K (*p* < 0.01), Mg (*p* < 0.01), and Ca (*p* < 0.01). K was positively correlated with Zn (*p* < 0.01) and Mg (*p* < 0.05). Mg was positively correlated with K (*p* < 0.05), Zn (*p* < 0.01), and Ca (*p* < 0.05).

**Table 2:** Correlation between the concentrations of ions in the egg yolk.

**Items**	**Cd**	**Pb**	**Cr**	**Hg**	**Se**	**Cu**	**Fe**	**Mn**	**Zn**	**Na**	**K**	**Mg**	**Ca**
Cd	1												
Pb	0.802^**^	1											
Cr	0.349	0.315	1										
Hg	0.524^*^	0.264	0.558^*^	1									
Se	−0.045	0.152	0.176	−0.112	1								
Cu	−0.263	−0.156	0.252	0.320	0.120	1							
Fe	0.510^*^	0.367	0.149	0.244	−0.155	−0.422	1						
Mn	−0.461	−0.584^**^	−0.091	−0.094	−0.500^*^	−0.045	0.026	1					
Zn	−0.119	−0.247	0.381	0.362	−0.323	0.680^**^	−0.084	0.371	1				
Na	0.017	0.386	0.161	−0.040	−0.160	−0.130	−0.038	−0.016	−0.154	1			
K	0.001	−0.156	0.307	0.449	−0.312	0.396	0.149	0.284	0.604^**^	0.258	1		
Mg	0.050	−0.237	0.139	0.209	−0.358	0.342	−0.137	0.365	0.624^**^	−0.308	0.459^*^	1	
Ca	0.130	−0.141	0.417	0.461	−0.120	0.580^**^	−0.182	0.010	0.686^**^	−0.161	0.394	0.475^*^	1

* *Measurements are significantly correlated at P < 0.05*;

** *measurements are significantly correlated at P < 0.01 (two-tailed)*.

#### Egg White

There were 10 significant correlations among the concentration of 13 elements in the egg white (Table 3). Cd was negatively correlated with Fe (*p* < 0.05) and Mg (*p* < 0.01). Pb was negatively correlated with Na (*p* < 0.05). Se was negatively correlated with Fe (*p* < 0.05). Mn was positively correlated with Zn (*p* < 0.05) and Mg (*p* < 0.05). K was positively correlated with Cu (*p* < 0.01), and Na (*p* < 0.01). Zn was positively correlated with Fe (*p* < 0.05) and Mn (*p* < 0.05). Mg was negatively correlated with Cd (*p* < 0.01) but positively correlated with Fe (*p* < 0.05) and Mn (*p* < 0.05).

**Table 3:** Correlation between the concentrations of ions in the egg white.

**Items**	**Cd**	**Pb**	**Cr**	**Hg**	**Se**	**Cu**	**Fe**	**Mn**	**Zn**	**Na**	**K**	**Mg**	**Ca**
Cd	1												
Pb	0.252	1.000											
Cr	0.018	0.138	1										
Hg	0.139	0.075	0.405	1									
Se	0.219	0.036	0.001	−0.295	1								
Cu	−0.283	−0.098	−0.030	−0.157	−0.204	1							
Fe	−0.688^*^	−0.210	−0.117	0.085	−0.588^*^	0.424	1						
Mn	−0.081	0.270	0.117	−0.010	−0.332	0.213	0.202	1					
Zn	0.067	0.394	0.079	0.433	−0.437	−0.062	0.564^*^	0.575^*^	1				
Na	0.088	−0.523^*^	−0.339	−0.157	0.040	0.350	0.125	−0.087	0.004	1			
K	0.039	−0.314	0.192	0.026	−0.164	0.592^**^	0.372	−0.014	0.144	0.571^**^	1		
Mg	−0.742^**^	−0.010	0.071	−0.142	−0.442	0.445	0.541^*^	0.557^*^	0.460	0.086	0.335	1	
Ca	−0.202	0.001	0.060	0.195	−0.126	0.098	−0.148	−0.293	−0.205	−0.156	−0.295	−0.066	1

* *Measurements are significantly correlated at P < 0.05*;

** *measurements are significantly correlated at P < 0.01 (two-tailed)*.

#### Ovary

There were 12 significant correlations among the concentration of 13 elements in the ovary (Table 4). Cd was positively correlated with Pb (*p* < 0.01) and Hg (*p* < 0.05) but negatively correlated with Cu (*p* < 0.05). Pb was positively correlated with Cd (*p* < 0.01), Hg (*p* < 0.05), and Fe (*p* < 0.05). Cr was correlated positively with Hg (*p* < 0.05), and negatively correlated with Mg (*p* < 0.05). Hg had a negative correlation with Cu (*p* < 0.05) and was positively correlated with Fe (*p* < 0.01), Cr (*p* < 0.05), Pb (*p* < 0.05), and Cd (*p* < 0.01). Cu was negatively correlated with Cd (*p* < 0.05) and Hg (*p* < 0.05), and was positively correlated with Ca (*p* < 0.05). Fe was positively correlated with Pb (*p* < 0.05) and Hg (*p* < 0.05), and negatively correlated with Zn (*p* < 0.05). K was positively correlated Mg (*p* < 0.05). Mg was positively correlated with K (*p* < 0.01), and negatively correlated with Cr (*p* < 0.05). Ca was positively correlated Cu (*p* < 0.05).

**Table 4:** Correlation between the concentrations of ions in the ovary.

**Items**	**Cd**	**Pb**	**Cr**	**Hg**	**Se**	**Cu**	**Fe**	**Mn**	**Zn**	**Na**	**K**	**Mg**	**Ca**
Cd	1												
Pb	0.665^*^	1											
Cr	0.315	0.252	1										
Hg	0.689^**^	0.553^*^	0.584^*^	1									
Se	0.028	−0.407	−0.300	−0.342	1								
Cu	−0.547^*^	−0.306	−0.127	−0.502^*^	−0.325	1							
Fe	0.425	0.585^*^	0.235	0.640^**^	−0.146	−0.371	1						
Mn	−0.163	0.357	0.104	−0.154	0.016	0.347	−0.249	1					
Zn	−0.341	−0.012	−0.023	−0.294	−0.145	0.114	−0.472^*^	−0.068	1				
Na	0.314	0.420	0.052	0.414	−0.173	−0.390	0.129	−0.408	0.189	1			
K	−0.165	−0.229	−0.235	−0.223	−0.147	0.230	0.016	−0.312	0.341	−0.126	1		
Mg	−0.279	−0.376	−0.457^*^	−0.161	−0.192	0.232	−0.120	−0.247	0.321	0.210	0.768^**^	1	
Ca	−0.426	−0.232	0.087	−0.332	−0.133	0.521^*^	−0.278	0.214	0.353	−0.203	0.039	0.042	1

* *Measurements are significantly correlated at P < 0.05*;

** *measurements are significantly correlated at P < 0.01 (two-tailed)*.

#### Oviduct

There were 16 significant correlations among the concentration of 13 elements in the oviduct (Table 5). Cd was positively correlated with Hg (*P* < 0.01) and Na (*P* < 0.01), and was negatively correlated with Mg (*p* < 0.01). Pb was positively correlated with Ca (*P* < 0.05). Cr was positively correlated with Fe (*p* < 0.05). Hg was positively correlated with Cd (*p* < 0.01), Zn (*p* < 0.05), and Na (*p* < 0.05), and was negatively correlated with Mg (*p* < 0.05). Cu was positively correlated with Mn (*p* < 0.01) and Zn (*p* < 0.01). Fe was positively correlated (*p* < 0.05) with Cr (*p* < 0.05) and Na (*p* < 0.01), and was negatively correlated with Mg (*p* < 0.05). Mn was positively correlated with Cu (*p* < 0.01), Zn (*p* < 0.01), and Ca (*p* < 0.05). Zn was positively correlated with Hg (*p* < 0.05), Cu (*p* < 0.01), Mn (*p* < 0.01), and Na (*p* < 0.01). Na was negatively correlated with Mg (*p* < 0.01), and positively correlated with Cd, Hg, Fe, and Zn (*p* < 0.05).

**Table 5:** Correlation between the concentrations of ions in the oviduct.

**Items**	**Cd**	**Pb**	**Cr**	**Hg**	**Se**	**Cu**	**Fe**	**Mn**	**Zn**	**Na**	**K**	**Mg**	**Ca**
Cd	1												
Pb	−0.170	1											
Cr	−0.034	−0.230	1										
Hg	0.949^**^	−0.105	−0.065	1									
Se	0.410	0.313	0.217	0.258	1								
Cu	−0.045	0.169	−0.302	0.317	0.261	1							
Fe	0.334	0.047	0.503^*^	0.444	0.083	0.300	1						
Mn	−0.134	0.167	−0.399	0.213	0.242	0.918^**^	−0.396	1					
Zn	0.441	0.069	−0.144	0.482^*^	0.030	0.614^**^	0.377	0.581^**^	1				
Na	0.735^**^	−0.106	0.307	0.540^*^	−0.101	−0.013	0.588^**^	−0.114	0.598^**^	1			
K	0.116	−0.052	0.218	0.232	−0.034	−0.100	0.183	−0.103	0.417	0.394	1		
Mg	−0.685^**^	0.127	−0.145	−0.514^*^	0.158	0.175	−0.542^*^	0.369	−0.402	−0.676^**^	−0.346	1	
Ca	−0.011	0.566^*^	−0.087	0.022	0.251	0.109	0.091	0.476^*^	0.182	0.160	0.144	0.286	1

* *Measurements are significantly correlated at P < 0.05*;

** *measurements are significantly correlated at P < 0.01 (two-tailed)*.

#### Follicular Walls

There were 14 significant correlations among the concentration of 13 elements in the follicular walls (Table 6). Cd was positively correlated with Pb (*p* < 0.01) and Hg (*p* < 0.05). Pb was positively correlated with Cd (*p* < 0.01), Hg (*p* < 0.01), and Fe (*p* < 0.05), but negatively correlated with Mn (*p* < 0.01). Hg was positively correlated with Fe (*p* < 0.05), Cd (*p* < 0.05), and Pb (*p* < 0.01). Se was negatively correlated with Mn (*P* < 0.05). Cu was positively correlated with Zn (*p* < 0.01) and Ca (*p* < 0.01). Zn was positively correlated with Cu (*p* < 0.01), K (*p* < 0.01), Mg (*p* < 0.01), and Ca (*p* < 0.01). K was positively correlated with Zn (*p* < 0.01) and Mg (*p* < 0.05). Mg was positively correlated (*p* < 0.01) with Zn (*p* < 0.05), K, and Ca (*p* < 0.05).

**Table 6:** Correlation between the concentrations of ions in the Follicle walls.

**Items**	**Cd**	**Pb**	**Cr**	**Hg**	**Se**	**Cu**	**Fe**	**Mn**	**Zn**	**Na**	**K**	**Mg**	**Ca**
Cd	1												
Pb	0.793^**^	1											
Cr	0.399	0.315	1										
Hg	0.631^*^	0.760^**^	0.067	1									
Se	−0.030	0.155	0.176	0.417	1								
Cu	−0.271	−0.155	0.254	−0.035	0.127	1							
Fe	0.356	0.542^*^	0.180	0.580^*^	−0.252	0.041	1						
Mn	−0.448	−0.586^**^	−0.091	−0.460	−0.499^*^	−0.050	0.114	1					
Zn	−0.124	−0.247	0.381	−0.178	−0.323	0.678^**^	0.273	0.373	1				
Na	0.039	0.388	0.161	0.215	−0.160	−0.132	0.152	−0.016	−0.154	1			
K	−0.030	−0.155	0.307	0.005	−0.312	0.394	0.325	0.286	0.604^**^	0.258	1		
Mg	0.010	−0.237	0.139	0.024	−0.358	0.340	0.350	0.366	0.623^**^	−0.308	0.459^*^	1	
Ca	0.146	−0.141	0.417	−0.077	−0.120	0.580^**^	−0.145	0.009	0.686^**^	−0.161	0.394	0.475^*^	1

* *Measurements are significantly correlated at P < 0.05*;

** *measurements are significantly correlated at P < 0.01 (two-tailed)*.

### Canonical Correlation Between Heavy Metal Elements in the Egg Yolk and Ovary

The first canonical correlation coefficient between the heavy metal concentration in the egg yolk and the ovary was 1.000 (Table 7) which reached a significant level (*p* = 0.03). The result indicated that there were positive correlations between the concentration of HEM in the ovary and in the egg yolk. Which one of HEM exists a positive correlation between the ovary and in the egg yolk? In order to find answers, the coefficient of X or Y in the first typical variables V_1_ and W_1_ regression equation would need to be judged. The composition of V_1_ and W_1_ is shown in Table 8. When the absolute coefficient before X or Y was simultaneously more than 0.5, the correlation of elements between egg yolk and ovary is supposed to exist. According to this rule, the concentration of Pb and Hg in the ovary was significantly positively correlated (*p* < 0.05) with those in the egg yolk.

**Table 7:** Typical correlation between heavy metal elements in the egg yolk and the ovary.

**Canonical variates**	**Canonical correlation coefficient**	**Eigen value**	**Wilk's^a^**	**DF^b^**	***P*-value**
1	1.000	1431.1	0.000	5.330	0.0320
2	0.989	43.59	0.007	1.684	0.2528
3	0.762	1.385	0.328	0.481	0.8497
4	0.455	0.262	0.782	0.262	0.8943
5	0.117	0.014	0.986	0.069	0.8030

a *Wilk's: A statistic for the ratio of two generalized variances*.

b *DF, Degree of Freedom*.

**Table 8:** Composition of the first pair of typical variables of heavy metal concentration in the egg yolk and ovary.

**Item**	**The composition of canonical variates**
Heavy metal in egg yolk	V_1_ = 0.130X_1_-0.500X_2_+0.357X_3_-0.788X_4_+0.267X_5_
Heavy metal in ovary	W_1_ = 0.517Y_1_-0.917Y_2_+0.563Y_3_-0.721Y_4_+0.027Y_5_

### Canonical Correlation Between Heavy Metal Elements in the Egg White and Viduct

The first canonical correlation coefficient between the heavy metal concentration in the egg white and in the oviduct was 0.9999 (*p* = 0.006) (Table 9). The result indicated that there were positive correlations between the concentration of HEM in the oviduct and those in the egg white. According to the aforementioned rule, the concentration of Cd in the egg white was significantly positively correlated (*p* < 0.05) with that in the oviduct (Table 10).

**Table 9:** Typical correlation between heavy metal elements in the egg white and heavy metal concentration in the oviduct.

**Canonical variates**	**Canonical correlation coefficient**	**Eigen value**	**Wilk's**	**DF**	***P*-value**
1	0.9999	6886.110	0.000	11.263	0.006
2	0.986	36.247	0.002	2.631	0.104
3	0.915	5.156	0.088	1.419	0.323
4	0.549	0.432	0.542	0.716	0.604
5	0.473	0.288	0.776	1.441	0.284

**Table 10:** Composition of the first pair of typical variables of heavy metal concentration in the egg white and the oviduct.

**Item**	**The composition of canonical variates**
Heavy metal in egg white	V_1_=0.932X_1_-0.329X_2_-0.136X_3_+0.469X_4_+0.283X_5_
Heavy metal in oviduct	W_1_=0.611Y_1_+0.378Y_2_-0.075Y_3_+0.158Y_4_+0.304Y_5_

## Discussion

In this study, we found that the complete feed contaminated with the combination of relatively higher levels of Cd, Cr, Pb, and Hg increased the Pb, Cd, and Hg deposition in the egg yolk and ovary, as well as the accumulation of Cd and Hg in the oviduct and the follicular wall. These results suggested that the ovary was the target reproductive tissue of the Cd, Pb, and Hg, and the oviduct and follicular wall were the special target reproductive tissues of Cd and Hg in laying hens. In agreement, dietary contamination of Cd, Pb, or Hg individual increased its deposition in the egg yolk (7, 9, 32, 33) and the ovary of hens (10, 12). Moreover, the Cd deposition in the ovary of laying hens increased in a dose-response manner with increasing dietary Cd level up to 210 mg/kg. However, the result of herein that dietary HEM didn't increase Cd, Pb, and Hg deposition in the egg white by dietary metals contamination was inconsistent with previous findings (7, 32, 33). Cappon and Smith (32) reported that dietary Hg administration at 13 mg/kg considerably increased the residue of Hg in the egg white of laying hens. Jeng et al. (7) found that dietary Pb inclusion (10 or 20 mg/kg body weight) increased Pb deposition in the egg white of laying ducks. Ma et al. (33) found that the accumulation of Hg in the egg white increased with the increasing dietary dosage of Hg from 0.28 to 27.24 mg/kg in hens aged 41 to 50 weeks. The discrepancies in these studies might be attributed to the differences in the mineral sources, the mineral dosages, the mode of applications, and the duration of mineral exposure.

The homeostasis of trace elements is essential to the normal operation of animal biochemical processes. Cell structure integrity, enzyme activity, and signal transduction are all based on the balance of trace elements (11, 18). Our study found that the HEM treatment increased the Fe concentration in the yolk, ovary, and oviduct, and decreased the Mn concentration in the yolk, the concentrations of Fe, Mn, and Zn in the egg white, the Cu concentration in the ovary, the concentration of Mg in the oviduct, and the concentrations of Cu, Zn, Mg, and Ca in the follicle wall. These results suggested the HME contamination caused the aforementioned ions disorder in the reproductive tissues of hens. Similarly, Li et al. (12) reported that feeding the diet added with 150 mg/kg CdCl_2_ for 90 days decreased the concentrations of Cu, Zn, and Ca, but increased Fe concentration in the ovary of hens aged 31 weeks. Qu et al. (13) found that Cd at 150 mg/kg decreased the concentration of Se in the breast muscle of hens. Why did the HEM treatment change other metals' retention in different tissues of hens? There were at least three reasons the following. First, the Cd and Pb have powerful interactions with other divalent metals, especially at the absorptive level (34). Gunshin et al. (35) also found that most of the divalent metal ions, including Cd^2+^, Pb^2+^, Zn^2+^, Mn^2+^, Cu^2+^, and Fe^2+^, shared the same transporter in the intestine of animals. Therefore, the changes in Zn, Mn, Cu, and Fe ions hemostasis were at least partly due to the competition of these metals with Cd and Pb in intestinal absorption. Second, the uptake of Cd from the intestine is assisted by metallothionein (MT), and Cd may replace the essential metals Cu and Zn or share the MT with them (34). The present result of the alteration of Cu and Zn balance was also caused by the MT binding competition of these metals in enterocytes. Third, excessive dietary Cd, Pb, and Hg could cause renal damage (34), which depressed the conversion of 25-hydroxyvitamin D to 1,25-dihydroxyvitamin D, and the renal reabsorption of Mg^2+^. Thus, the potential damage of kidney of hens by HEM treatment might have caused the disorder of Ca and Mg ions hemostasis in the oviduct and follicle walls.

The present study revealed 42 unique correlations between metal elements in the ovary, oviduct, and follicle wall, and Pearson's correlation coefficients showed the positive correlations among the Cd, Pb, Hg, and Cr retention but the negative correlations between the Cd, Pb, Hg, Cr, and Mg, Cu, Zn. These results indicated the heavy metals Cd, Pb, Hg, and Cr had a synergistic effect on their retention, but they also had a negative effect on the Mg, Cu, and Zn deposition in different reproductive tissues. In agreement, excessive dietary Hg exposure combined with Cd and Pb elevated the deposition of Hg in the liver of Peking ducks (36). Chen et al. (6) reported that the combined 100 mg/L Pb and 50 mg/L Cd stimulated the deposition of Pb and Cd in the liver of laying hens, suggesting that Pb and Cd have a synergistic effect on the liver of laying hens. Our correlation analyses also proved the HEM treatment caused the Mg, Cu, and Zn disorder in the reproductive tissue. In the present study, the HEM treatment increased the Fe retention in the oviduct and ovary. Similarly, Zhang et al. (2017) and Li et al. (12) also found that excessive Cd increased the concentration of Fe in the kidneys or ovary of laying hens. These results supported and strengthened our findings herein. It was possible that the HEM treatment caused tissue injury and increased blood flow and hemolysis, resulting in an increase in Fe retention. The increase in Fe concentration also may be due to the rupture of red blood cells and the influx of Fe^2+^ into the ovary or oviduct. Unfortunately, we did not determine the concentration of heme-iron, and future work should focus on the changes in different forms of iron in the tissues.

In this study, dietary Se supplementation did not change the concentrations of Cd, Pb, Hg, and Cr in the egg yolk, ovary, oviduct, and follicle wall in hens fed the diet with these heavy metals contamination, although it increased the concentration of Se in the egg white, egg yolk, oviduct, and follicular wall. These results suggested the Se addition did not affect the heavy metals deposition in the reproductive organs or tissues. These results were consistent with the previous studies about the alleviating effect of Se on the Cd individual toxicity (12, 37). Li et al. (12) found that the addition of 2 mg/kg of Na_2_SeO_3_ in diet did not reduce the increase of Cd deposition in the ovary of hens caused by dietary addition of 150 mg/kg CdCl_2_. Al-Waeli et al. (37) also reported that Se at 3 mg/kg has no significant influence on the Cd deposition in the kidney and liver of broilers when 100 mg Cd /kg was supplemented with the diet. In contrast, Zhang et al. (14, 38, 39) found that Se at 2 mg/kg ameliorated deposition of Cd in the kidneys, liver and bone of laying hens by dietary addition of 150 mg /kg Cd. The discrepancies in these findings might be due to the differences in the selected tissues, the mineral dosages, Se form, and poultry species.

Additionally, we also found that dietary Se addition altered the Mg ion hemostasis in the oviduct and follicle walls, and Fe concentration in the oviduct of hens. These results demonstrated that dietary Se addition at 0.4 mg/kg had an ameliorative effect on the Mg and Fe ions disorder but had no restoring effect on the other ions disorder in hens caused by HEM treatment. However, Xu et al. (20) found that dietary addition of 0.2 mg/kg Se aggravated the decreases of Cu and Mn in the liver of broilers induced by 0.5 mg/kg Pb exposure. Several studies reported that dietary Se addition alleviated the Fe, Cu, Mn, and Zn ions disorder in the liver, kidney, and muscle of broilers or hens caused by excessive Cd (12, 14, 38, 40). The differences of one heavy metal individual or several heavy metals combination exposure could partly explain the Se is an essential microelement in animals, and plays an important role in the antioxidant defense process (8). Dietary Se supplementation increased the activities of the superoxide dismutase and glutathione peroxidases in the ovary of hens with excessive Cd exposure (12). Similar finding was observed in the kidney of chickens (14). Therefore, we speculated that the alleviating effect of Se supplementation on the Fe and Mg ions disorder in oviduct and follicle walls was partly due on its protective effect on the renal damage of hens caused by HEM treatment, resulting in the reabsorption of Mg^2+^ and excretion of Fe^2+^.

Nad et al. (8). The effect of cadmium in combination with zinc and selenium on ovarian structure in Japanese quails.

The follicle wall consists of the inner basement membrane, granular cells, and perivitelline radial bands, which regulate the export of trace minerals to the embryo during development (41, 42). In the present study, the results of CCA analyses showed that there was a positive relationship in the heavy metals retention between the ovary and yolk, and the oviduct and egg white. Egg yolk and egg white come from the ovary and oviduct of hens, respectively; each yolk is enclosed in a follicle, attached to the ovary (43, 44). These results of CCA analyses also suggested the nutrients transferred from the ovary to the yolk, and from the oviduct to the egg white. The composition of the first pair of canonical correlation variates showed that, for the excessive Cd, Pb, Hg, and Cr exposed-hens, the Pb and Hg retention played the vital roles in other heavy metals deposition in the ovary and egg yolk, respectively; the Cd retention played the important role in the other heavy metals deposition in the oviduct and egg white. These results suggested that one heavy metal is mostly deposited in some special tissues, although they have a synergistic effect on their retention. However, our study only focused on the responses of different heavy metals in combination, and the role of each heavy metal remains to be determined in the future.

## Conclusion

Excessive dietary Cd, Pb, Hg, and Cr in combination increased the deposition of Cd, Pb, and Hg, and disturbed ion hemostasis in the different reproductive organs or tissues of laying hens. Dietary Se addition at 0.4 mg/kg from selenized yeast alleviated the negative effects of HEM on Fe and Mg ion disorder in the oviduct and follicle wall of hens.

## Data Availability Statement

The original contributions presented in the study are included in the article/supplementary material, further inquiries can be directed to the corresponding author.

## Ethics Statement

The animal study was reviewed and approved by the experiment was performed following the Chinese Guidelines for Animal Welfare set by the National Institute of Animal Health and approved by the Animal Care and Use Committee of Sichuan Agricultural University.

## Author Contributions

CW, SB, KL, and KZ: conceptualization. LL, GL, SB, JW, and YL: methodology. LL, YJ, JS, RZ, FW, and BW: investigation. CW, SB, LL, BW, and YJ: formal analysis. CW, LL, and SB: writing-original draft preparation and data curation. GL, KZ, JW, and WK: supervision. SB, CW, and KL: funding acquisition. All authors reviewed, edited, and approved the final version of the manuscript. All authors contributed to the article and approved the submitted version.

## Funding

This work was supported by the National Key Research and Development Program of China (No. 2021YFD1300203).

## Conflict of Interest

BW is employed by Chelota biotechnology Co., Ltd. The remaining authors declare that the research was conducted in the absence of any commercial or financial relationships that could be construed as a potential conflict of interest.

## Publisher's Note

All claims expressed in this article are solely those of the authors and do not necessarily represent those of their affiliated organizations, or those of the publisher, the editors and the reviewers. Any product that may be evaluated in this article, or claim that may be made by its manufacturer, is not guaranteed or endorsed by the publisher.
